# Use of the Bruininks-Oseretsky test of motor proficiency (BOT-2) to assess efficacy of velmanase alfa as enzyme therapy for alpha-mannosidosis

**DOI:** 10.1016/j.ymgmr.2020.100586

**Published:** 2020-04-08

**Authors:** Dawn Phillips, Julia B. Hennermann, Anna Tylki-Szymanska, Line Borgwardt, Mercedes Gil-Campos, Nathalie Guffon, Yasmina Amraoui, Silvia Geraci, Diego Ardigò, Federica Cattaneo, Allan M. Lund

**Affiliations:** a UNC Chapel Hill, Division of Physical Therapy, School of Medicine, Chapel Hill, NC, United States of America; b University Medical Centre Mainz, Dept. Pediatric and Adolescent Medicine, Villa Metabolica, Mainz, Germany; c Department of Paediatric, Nutrition and Metabolic Diseases, The Children's Memorial Health Institute, Warsaw, Poland; d Centre for Inherited Metabolic Diseases, Department of Paediatrics and Department of Adolescent Medicine, Copenhagen University Hospital, Rigshospitalet, Copenhagen, Denmark; e Center for Genomic Medicine, Copenhagen University Hospital, Rigshospitalet, Copenhagen, Denmark; f Metabolism and Pediatric Research Unit, Reina Sofia University Hospital, IMIBIC, University of Cordoba, Unidad de Metabolismo e Investigación Pediátrica, Hospital Universitario Reina Sofía, Universidad de Córdoba, CIBERObn, Córdoba, Spain; g Reference Center for Inherited Metabolic Diseases, Femme Mere Enfant Hospital, Lyon, France; h SphinCS GmbH, Clinical Science for LSD, Hochheim, Germany; i Chiesi Farmaceutici S.p.A, Parma, Italy

## Abstract

**Objectives:**

Alpha-mannosidosis is a rare autosomal recessive lysosomal storage disorder resulting from deficient lysosomal alpha-mannosidase activity. Clinical manifestations include progressive balance disorders, immune deficiency, skeletal abnormalities and cognitive deficits beginning in early childhood. Enzyme replacement therapy with recombinant human alpha-mannosidase (velmanase alfa) is scheduled for clinical development in the US beginning in 2020 and has been approved in the EU for treatment of non-neurological manifestations in cases of mild to moderate disease. This study assessed effects of velmanase alfa on fine and gross motor proficiency in children and adults.

**Methods:**

Integrated Bruininks-Oseretsky (BOT-2) test of Motor Proficiency data from velmanase alfa clinical trials was stratified by age for 14 adults and 19 children treated for up to 4 years.

**Results:**

Patients showed global developmental delays at baseline. For the combined adult and pediatric group there was a statistically significant increase (improvement) in BOT-2 total point score of 13% (p = .035, 95% CI 1.0, 25.0) from baseline to last observation. When stratified by pediatric versus adult patients, there was improvement in BOT-2 total point score in patients <18 years (mean percent increase from baseline to last observation 23%) compared to adults (mean decrease of −0.7%). Subtest analysis of individual BOT-2 items captured some improvement following velmanase alfa treatment in pediatric patients.

**Conclusions:**

There was limited ability to assess the BOT-2 change responses in adults. Pediatric patients showed stability or improvement in scaled scores relative to healthy peers, indicating continued skill acquisition, which may increase independence and contribute to improved patient quality of life.

## Highlights

• Alpha-mannosidosis patients have global developmental delay, requiring assistance with many activities of daily living.
• BOT-2 is used to assess improvements in functional capacity in children and young adults.
• Pediatric patients showed stability or improvement in BOT-2 scaled scores relative to healthy peers, indicating continued skill acquisition.

## 1 Introduction/background

Alpha-mannosidosis (OMIOM 248500) is a rare autosomal recessive lysosomal storage disorder caused by pathogenic variants in the *MAN2B1* gene, resulting in a deficiency of lysosomal alpha-mannosidase activity (EC3.2.1.24). Alpha-mannosidase is responsible for degradation of N-linked oligosaccharides, and its deficiency results in progressive accumulation of mannose-rich oligosaccharides in all tissues, with multi-systemic disease arising from impaired cell function and cell death in visceral and CNS tissues [1]. Diagnosis is confirmed by increased levels of mannose-rich oligosaccharides in urine, reduced enzyme activity in serum/leukocytes, and genetic testing that identifies the pathogenic variants on both alleles of the *MAN2B1*gene, which may be identical (homozygous) or different (compound heterozygous). Birth prevalence is estimated at 1 in 300,000 to 1,000,000 depending on region, and is panethnic [1].

Natural history data from cross-sectional and longitudinal studies [2,3] as well as from a small number of survey or case studies [[4], [5], [6]] have demonstrated disease heterogeneity and significant disease burden. A clinical spectrum of symptoms and disease progression is seen with alpha-mannosidosis, ranging from mild to severe [2], with severity impacted in part by level of residual alpha-mannosidase activity [7] and subcellular localization of mutant *MAN2B1* protein [8]. While most children appear normal at birth, clinical manifestations such as hearing loss begin at an early age followed by progressive development of skeletal abnormalities (including osteopenia, calvaria, vertebrae deformation, genu valgum, and joint deterioration), balance disorders, immune deficiency and cognitive deficits [2,3]. Mild forms progress more slowly depending on age of onset [7] while severe disease is associated with death in early childhood from CNS involvement or infections. The long-term prognosis for untreated alpha-mannosidosis is poor due to cognitive, neuromuscular, and skeletal deterioration that lead to loss of independence in mobility and activities of daily living (ADLs) and chronic pain [3,9]. Assessment of cognitive and functional ability of 35 individuals with attenuated alpha-mannosidosis age 6–35 years show that individuals have mild to severe intellectual disability (IQ of 30–81) that remains stable from adolescence, as well as variable difficulties with visual function, reasoning, and visuospatial skills [5]. In addition to cognitive and intellectual decline, motor function deteriorates over time with ataxia, abnormal balance (in the absence of true ataxia), hypotonia, and increase in pain [2,6]. A longitudinal study of 43 individuals with alpha-mannosidase reported that clinical abnormalities in the musculoskeletal system were present from early childhood that impacted mobility and physical endurance in adolescence and adulthood [3].

Treatment options for alpha-mannosidosis are limited. Allogeneic hematopoietic stem cell transplantation (HSCT) has been used to preserve neurocognitive function, improve general status, and prevent early death [[10], [11], [12]], although a better understanding of HSCT timing and regimens as well as impact on outcomes is needed [10,13]. Enzyme replacement therapy (ERT) with a recombinant human alpha-mannosidase (velmanase alfa) is scheduled for clinical development in the US beginning in 2020 and has been approved in the EU for treatment of non-neurological manifestations in individuals with mild to moderate disease. Phase 1/2 and 3 clinical trials of velmanase alfa have demonstrated sustained decrease in serum oligosaccharides and improvements in physical endurance. [14,15]. Long-term integrated analysis of clinical trial safety and efficacy data have shown that improvements in biochemical and functional measures were sustained for up to 4 years, with better functional improvements related to initiation of treatment at an early age [16]. Post-hoc analysis using a global treatment response endpoint incorporating pharmacodynamic, functional, and quality of life measures showed that 87% of individuals receiving velmanase alfa were treatment responders compared to 30% receiving placebo [17].

The Bruininks-Oseretsky test of Motor Proficiency - 2nd Edition (BOT-2) is used to assess improvements in functional capacity in children and young adults with differentiated measures of gross and fine motor proficiency. The BOT-2 is a standardized, normative assessment (including age- and gender-specific norms) for individuals 4–21 years of age and can be extended to use beyond 21 years without normative data. Although availability of BOT-2 assessments varies, it was administered during clinical development of velmanase alfa to assess proficiency in fine motor control, manual coordination, running speed and agility. Integrated analysis of patients enrolled in velmanase alfa trials showed that BOT-2 total point scores increased from baseline to last observation, and the percent change from baseline was statistically significant (p = .035) [16]. In the previous publication, however, results were not stratified by age, and only total point scores for the study population were presented.

In order to better determine changes in fine and gross motor proficiency over time, raw BOT-2 subtest data can be normalized by age and gender and converted to age equivalent scores for the entire population, and scale scores for pediatric patients. The objectives of the current study were to analyze integrated BOT-2 data throughout velmanase alfa clinical development with normative and age-equivalent scores for pediatric and adult patients.

## 2 Methods

**Fig. 1:**
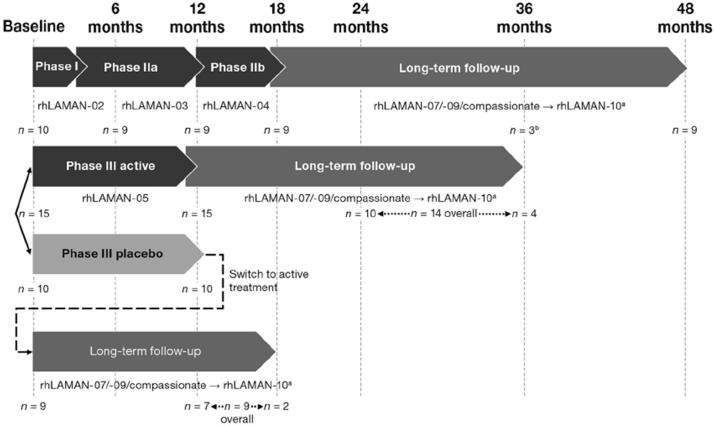
Numbers of patients and contributing studies to integrated analysis.

Data on BOT-2 and other functional outcomes were obtained from an integrated analysis of long-term safety and efficacy of velmanase alfa in 33 pediatric and adult patients with alpha-mannosidosis (rhLAMAN-10; NCT 02478840). All patients or caregivers provided informed consent. Individual patient data from previous phase 1, 2 or 3 velmanase alfa trials and extension studies were integrated into a single database for analyses. Data were included for patients receiving weekly intravenous infusions of 1 mg/kg velmanase alfa through either follow-on extension studies or compassionate use programs. Fig. 1 illustrates patient distribution and the contributing studies. Integrated analyses were evaluated in the overall population, in the adult cohort (≥18 years of age) and in the pediatric cohort (<18 years of age).

Baseline functional data were derived from the original clinical trials in which patients were enrolled. Baseline levels of disability and pain were assessed using the Childhood Health Assessment Questionnaire (CHAQ). Quality of life was assessed using the EuroQol 5 Dimension-5 Level Questionnaire (EQ-5D-5 L).

### 2.1 BOT-2 description and scoring

**Fig. 2:**
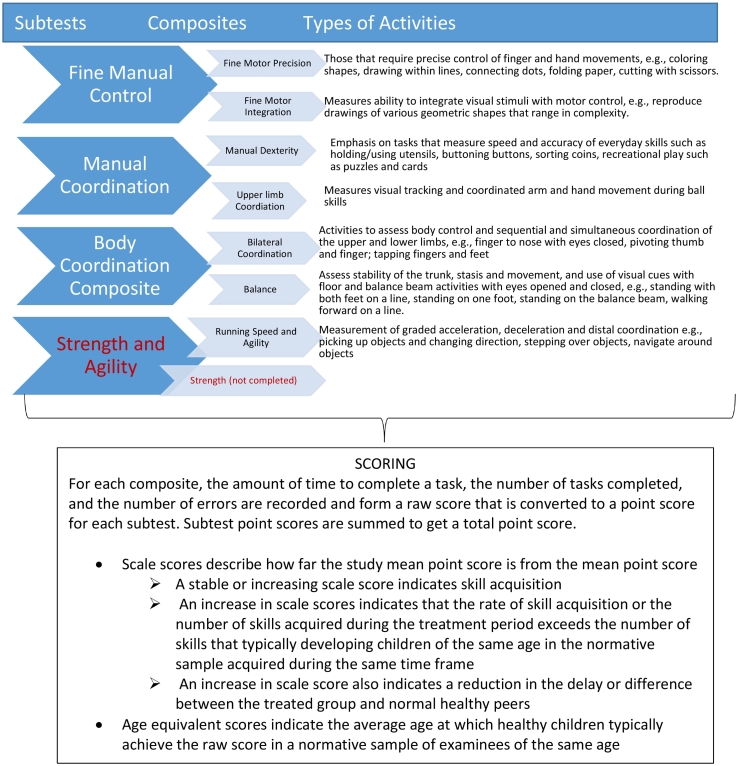
The BOT-2 test includes subtests for fine motor precision, fine motor integration. Manual dexterity, upper limb and bilateral coordination, balance, running speed and agility, and strength. Subset point scores can be used to derive scaled and age-adjusted scores in pediatric patients.

BOT-2 testing includes subtests for fine motor precision (FMP), fine motor integration (FMI). Manual dexterity (MD), upper limb coordination (ULC) and bilateral coordination (BC), balance, running speed and agility (RSA), and strength. Fig. 2 illustrates the structural organization of the composites and subtests of the BOT-2 test, and the approach to scoring. The FMP test requires precise control of finger and hand movements in tasks that include coloring shapes, drawing within lines, connecting dots, folding paper, and cutting with scissors. The FMI test requires patients to reproduce drawings of various geometric shapes and measures integration of visual stimuli with motor control. MD emphasizes tasks that measure fine motor speed and accuracy such as placing pegs into a peg board, sorting cards, transferring pennies, stringing blocks and accuracy of making dots within small circles. The items are designed to capture the dexterity required to complete everyday skills such as holding/using utensils, buttoning buttons, sorting coins, recreational play such as puzzles and cards. ULC is designed to measure visual tracking and coordinated arm and hand movement during ball skills. In BC assessment, tasks require body control and sequential and simultaneous coordination of the upper and lower limbs. Items include positioning finger to nose with eyes closed, pivoting the thumb to finger, tapping fingers and feet (with ipsilateral and contralateral extremity), jumping jacks, and jumping in place, (with ipsilateral and contralateral extremity coordination). The balance assessment involves three areas that affect balance: stability of the trunk, stasis and movement and use of visual cues. Stability of the trunk is measured with standing on both feet on a line, standing on one foot, standing on the floor and standing on the balance beam, movement items include walking forward on a line and three tasks require the patient to have eyes closed to examine the extent in which visual cues are used for balance. For the eyes open balance items, the patient is instructed to stand in the defined position, place hands on hips and look at a red circular target that has been placed at eye level on the wall that is used as a visual cue to help stabilize balance and maximize attention. For RSA, tasks include the shuttle run to assess measurements of acceleration and deceleration, picking up objects, and changing direction, stepping sideways over a balance beam to assess distal coordination, navigation around obstacles, and one and two-legged hopping.

Item performance on the BOT-2 is expressed in several ways, including time taken to complete a task, the number of tasks completed, and the number of errors occurring while completing a task. Each raw score on a task is converted to a point score to allow performance to be measured on a graded scale. The individual item points are summed to derive a subtest point score. The subtest point scores for seven subtests [(FMP, FMI, MD, ULC, BC, balance, and RSA) were summed to achieve a total subtest point score. An eighth subtest, Strength, was not evaluated and collected due to characteristics of the patient population that prevented completion of the strength items.

The subtest point scores were used to derive a scale score. Scale scores describe comparability to a normative sample of examinees of the same age and have a mean of 15 with a standard deviation of 5. BOT-2 scale score normative values are only available until 21 years of age and were used for the pediatric cohort (<18 years of age). A stable or increasing scale score indicates skill acquisition. An increase in scale score indicates that the rate of skill acquisition or number of skills acquired during the treatment period exceeds those for developing children in the normative sample of the same age during the same time frame.

Since scale scores were not available for the adult group, subtest total point scores were converted to age equivalent scores. In the normative sample, age equivalent scores indicate the average age at which healthy individuals typically achieve the raw score. An increase in age equivalent values indicates skill acquisition.

## 3 Results

Demographic characteristics have been presented previously [16,18]. Fourteen patients were in the adult cohort age 18–35 years, with a mean (SD) age of 24.6 (5.3) years. Nineteen patients were in the pediatric cohort with an age range 6–17 years at baseline. All patients were Caucasian, and 20/33 (61%) were male. Baseline characteristics are presented in Table 1.

**Table 1:** Baseline characteristics and assessments.

	Total Population	Pediatric Cohort <18 years	Adult Cohort ≥18 years
	N = 33	N = 19	N = 14
Mean Age (SD) years	17.1 (7.8)	11.6 (3.7)	24.6 (5.3)
Sex n (%)			
Female	13 (39.4)	6 (31.6)	7 (50)
Male	20 (60.6)	13 (68.4)	7 (50)
Assistance required for ambulation n (%)	10 (30.3)	5 (26.3)	5 (35.7)
6MWT meters mean (SD)	N = 33	N = 19	N = 14
	466.6 (90.1)	454.2 (86.3)	483.4 (95.6)
CHAQ-Disability Index mean (SD)	N = 33	N = 19	N = 14
	1.36 (0.77)	1.22 (0.89)	1.55 (0.55)
CHAQ-pain VAS mean (SD)	N = 31	N = 18	N = 14
	0.618 (0.731)	0.450 (0.508)	0.834 (0.920)
EuroQol-5D-5 L mean (SD)	N = 24	N = 10	N = 14
	0.622 (0.170)	0.697 (0.184)	0.568 (0.142)
Leiter-R mean (SD)	N = 33	N = 19	N = 14
	5.879 (1.565)	5.399 (1.399)	6.530 (1.590)
BOT-2 total point score mean (SD)	N = 29	N = 17	N = 12
	107.0 (47.6)	101.9 (53.8)	113.9 (38.6)

6MWT: distance that a patient can walk on a flat, hard surface in a period of 6 min.

Leiter: all values were displayed as age equivalent values which indicate the average age at which healthy children typically achieve the score.

CHAQ: Disability Index scores range from 0 to 3 with higher scores indicating greater disability. Discomfort was determined by the presence of pain measured on a 15 cm visual analogue scale (VAS) anchored at either end by 0 as “no pain” and 100 as “very severe pain”. The distance from the left hand side to the mark is then multiplied by 0.2 to obtain a value from 0 to 3.

EQ: measurement of health through 5 dimensions (Mobility, Self-care, Usual Activities, Pain/discomfort, and Anxiety/depression). Each dimension is rated on a 5 point scale from no problems (1) to extreme problems or unable to complete (5). The dimension scores create a single summary Health Index (range from 0 to 1). A higher EQ-5D-5 L Health Index score indicates better health.

BOT-2: maximum total point score 278.

Multi-systemic impairment was present and is described in Table 1. Overall, 30.3% (10/33) of patients required mobility assistance (help from a person, walking aids, or a wheelchair) at baseline. In the pediatric and adult cohorts, 26.3% (5/19) and 35.7% (5/14) of patients, respectively, required assistance. The overall population mean (SD) baseline CHAQ VAS pain score was 0.618 (0.731), and the adult group had a higher baseline mean [SD] level of pain (0.834 [0.920]) compared to the pediatric group (0.450 [0.508]). The mean (SD) EQ-5D-5 L health index values at baseline were 0.697 (0.184) for pediatric patients and 0.568 (0.142) for adults, with an overall population score of 0.622 (0.170). Impairment in multiple systems were observed in the majority of patients, including the nervous system (84.8%), auditory function (81.8%), and speech (78.8%). The visual system was impaired in 54.5% of patients. Psychiatric (81.8%) and musculoskeletal and connective tissue (60.6%) disorders were also present, and some patients experienced ataxia (24.2%).

Table 2, Table 3, Table 4, Table 5, Table 6, Table 7 show the baseline age equivalent and scale scores for adult and pediatric patients and indicate global developmental delays. Age equivalent baseline mean scores for pediatric patients ranged from 4.1 (on the Balance subtest) to 6.0 (FMP), and were well below the mean (SD) subject chronological age of 11.6 (3.7) years. For scaled scores among pediatric patients, the normative mean (SD) is 15 (5). Across the 7 subtests, baseline mean scale scores were > 2SD below the normative mean for 5 tests (Balance, MD, BC, ULC, RSA) approached 2SD below the normative mean (5.7 and 5.4, respectively) for FMI and FMP. The mean (SD) age of the adults was 24.6 (5.3) years with a mean age equivalent range of function from 4.0 years on the Running Speed and Agility to 7.8 years for Fine Motor Precision subtests.

**Table 2:** Fine motor precision.^a^

Group	Baseline	Last Observation	Absolute Change	Percent Change
	Mean (SD)	Mean (SD)	Mean (SD)	Mean (SD)
Overall N = 33 Age Equivalent	6.8 (2.0)	7.2 (2.4)	0.4 (1.6)	7.1 (21.0)
≥18 N = 14 Age Equivalent	7.8 (1.9)	7.7 (2.1)	−0.1 (1.1)	−0.7 (12.4)
<18 N = 19 Age Equivalent	6.0 (1.7)	6.7 (2.6)	0.8 (1.8)	12.9 (24.3)
Scale score	5.4 (2.3)	6.2 (4.1)	0.7 (3.2)	10.2 (45.9)

a Includes activities that require precise control of finger and hand movements, such as coloring shapes, drawing within a path, connecting dots, folding paper, cutting a circle. Scale score normative mean 15 (SD 5).

**Table 3:** Fine motor integration.^a^

Group	Baseline	Last Observation	Absolute Change	Percent Change
	Mean (SD)	Mean (SD)	Mean (SD)	Mean (SD)
Overall N = 33 Age Equivalent	6.5 (2.4)	6.6 (2.6)	0.2 (2.0)	5.1 (26.9)
≥ 18 N = 14 Age Equivalent	7.5 (2.7)	7.2 (2.7)	−0.3 (2.2)	−0.5 (24.4)
< 18 N = 19 Age Equivalent	5.7 (1.8)	6.2 (2.5)	0.5 (1.8)	9.2 (28.6)
Scale score	5.7 (3.6)	5.4 (3.8)	−0.4 (2.4)	−5.6 (41.4)

a Measures the ability to integrate visual stimuli with motor control and requires patients to reproduce drawings of various geometric shapes that range in complexity from a simple circle to overlapping pencils. Scale score normative mean 15 (SD 5).

**Table 4:** Manual dexterity.^a^

Group	Baseline	Last Observation	Absolute Change	Percent Change
	Mean (SD)	Mean (SD)	Mean (SD)	Mean (SD)
Overall N = 33 Age Equivalent	5.1 (1.1)	5.4 (1.2)	0.3 (0.8)	6.6 (16.6)
≥18 N = 14 Age Equivalent	5.0 (0.9)	5.1 (0.9)	0.1 (0.7)	3.1 (11.3)
<18 N = 19 Age Equivalent	5.2 (1.2)	5.6 (1.4)	0.4 (0.9)	9.3 (19.5)
Scale score	3.8 (2.1)	4.4 (2.5)	0.6 (1.6)	27.4 (4.2)

a Emphasis on speed and accuracy using tasks designed to correspond to activities of daily living such as holding and eating utensils, buttoning buttons, and sorting coins to make change as well as recreational play like puzzles and cards. Scale score normative mean 15 (SD 5).

**Table 5:** Upper limb coordination.^a^

Group	Baseline	Last Observation	Absolute Change	Percent Change
	Mean (SD)	Mean (SD)	Mean (SD)	Mean (SD)
Overall N = 33 Age Equivalent	5.9 (1.6)	6.1 (1.8)	0.2 (1.3)	0.2 (1.3)
≥ 18 N = 14 Age Equivalent	5.9 (1.4)	5.8 (1.2)	−0.2 (0.8)	−0.7 (12.7)
< 18 N = 19 Age Equivalent	5.8 (1.7)	6.3 (2.1)	0.5 (1.6)	10.7 (30.1)
Scale score	4.8 (2.5)	5.1 (3.6)	0.3 (2.8)	34.2 (167.4)

a Designed to measure visual tracking and coordinated arm and hand movement during ball skills. Scale score normative mean 15 (SD 5).

**Table 6:** Bilateral coordination.^a^

Group	Baseline	Last Observation	Absolute Change	Percent Change
	Mean (SD)	Mean (SD)	Mean (SD)	Mean (SD)
Overall N = 33 Age Equivalent	4.8 (1.0)	5.0 (1.0)	0.3 (0.7)	6.2 (13.1)
≥18 N = 14 Age Equivalent	4.8 (1.0)	4.9 (0.9)	0.1 (0.5)	3.2 (9.3)
<18 N = 19 Age Equivalent	4.8 (1.0)	5.2 (1.0)	0.4 (0.8)	8.5 (15.2)
Scale score	4.8 (1.8)	5.2 (1.3)	0.4 (1.3)	15.9 (32.7)

a Tasks require body control and sequential and simultaneous coordination of the upper and lower limbs. Scale score normative mean 15 (SD 5).

**Table 7:** Balance.^a^

Group and Score	Baseline	Last Observation	Absolute Change	Percent Change
	Mean (SD)	Mean (SD)	Mean (SD)	Mean (SD)
Overall N = 33 Age Equivalent	4.1 (0.2)	4.1 (0.4)	0.0 (0.4)	0.9 (9.4)
≥18 N = 14 Age Equivalent	4.0 (0)	4.0 (0)	0	0
<18 N = 19 Age Equivalent	4.1 (0.3)	4.1 (0.5)	0.1 (0.5)	1.6(12.5)
Scale score	2.8 (1.7)	2.7 (1.9)	−0.1 (1.7)	6.7 (51.7)

a Involves three areas that affect balance: stability of the trunk, stasis and movement and use of visual cues. Scale score normative mean 15 (SD 5).

**Fig. 3:**
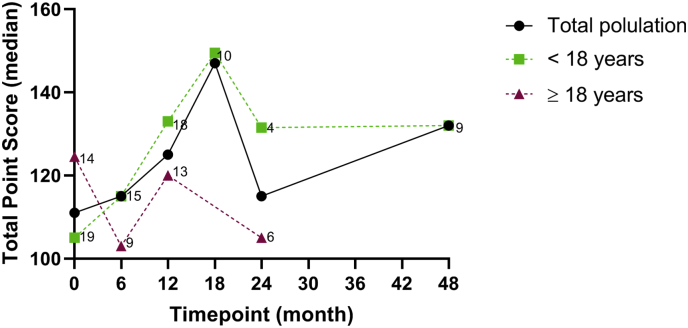
BOT-2 total point score by time point and age groups. The median BOT-2 total point score absolute values are plotted for the total population, and pediatric and adult groups for time points with greater than 2 observations (numbers next to symbols indicate number of observations).

All patients had at least 12 months of treatment data, 19 had at least 24 months, and pediatric patients (n = 9) from the original phase 1 and 2 trials had 48 months of data. Median BOT-2 total point scores for the overall population and for the pediatric and adult groups are shown in Fig. 3. Overall, for the combined adult and pediatric group there was a statistically significant increase in BOT-2 total point score at Month 12 (absolute change +7.5 p = .017 95% CI 1.4, 13.5); percent change 10.6% p = .005 95% CI 3.5, 17.7) and a statistically significant increase in BOT-2 total point score percent change from baseline to last observation (percent change 13.0% p = .035 95% CI 1.0, 25.0). The absolute change from baseline (+5.1 p = .23 95%CI -3.4, 13.6) at last observation did not reach statistical significance. When stratified by pediatric versus adult patients, there were fluctuations in BOT-2 total scores over time, but consistently greater changes from baseline were observed only in patients <18 years compared to adults.

Subtest age equivalent and scale scores were determined for the overall population and for the pediatric and adult groups. In the FMP test (Table 2), all subtest items had some patients that improved. The FMP mean Baseline point score was higher for adults (32.2) than for children (22.5). The paper folding item had the greatest number of adults (9/14, 64%) and children (13/19, 68%) demonstrating improvement, and improvement was similar in both groups. Nine of 14 (64%) and 10/14 (71%) of adults had baseline maximal scores on the cutting and filling in the circle items, respectively, indicating a ceiling effect that limited assessment of change.

In the FMI test, skill acquisition was seen on tasks, but at slower rates than in the normative peer sample (Table 3). Therefore the mean age equivalent score showed a small increase and the scale score showed a decrease. Table 4 shows results for MD, which requires goal directed activities that involve reaching, grasping and bimanual coordination and emphasizes speed and accuracy. Skill acquisition was seen with an increase in both the age equivalent and scale scores. Upper Limb Coordination (Table 5): Skill acquisition was also seen for ULC with an increase in both the age equivalent and scale scores.

The BC test includes several measures of ataxia that showed improvement or stability overall (Table 6). The finger to nose task improved or was stable in 28/33 (85%) patients. Simultaneous tapping with fingers and feet improved or was stable in 22/33 patients for the same side assessment and 27/33 for the opposite side assessment. There was an increase in both the age equivalent and scale scores, suggesting skill acquisition.

In general the majority of patients were challenged to complete many of the balance tasks and improvements were modest. For the easiest balance item, of standing on line with space between two feet, at Baseline 15/33 patients were unable to stand for 3 s with eyes open, and 25/33 were unable with eyes closed. Standing on one foot was even more challenging, with 23/33 and 33/33 patients unable to stand for 3 s with eyes open and closed, respectively. Seven patients had an improved ability to stand on one foot with eyes open and 11 patients improved standing on one foot with eyes closed at the last assessment.

For RSA (Table 8), some patients did have improvements on individual items, but the scoring reflects a floor effect on the shuttle run. All shuttle run times of ≥16 s are given a point score of 0, and improvements from 19 to 16 s would not equate to a change in point score. 18/33 patients had a reduction in the time to complete the shuttle run, and 6/18 patients had values of ≥16 that did not result in a change in point score. The majority of patients had difficulty with the unilateral hopping items.

**Table 8:** Running speed and agility.^a^

Group	Baseline	Last Observation	Absolute Change	Percent Change
	Mean (SD)	Mean (SD)	Mean (SD)	Mean (SD)
Overall N = 33 Age Equivalent	4.3 (0.5)	4.3 (0.7)	0.0 (0.5)	0.1 (9.3)
≥18 N = 14 Age Equivalent	4.1 (0.2)	4.1 (0.2)	−0.0 (0.1)	−0.2 (2.3)
<18 N = 19 Age Equivalent	4.5 (0.5)	4.5 (0.9)	0.0 (0.6)	0.4 (12.2)
Scale score	4.0 (2.3)	2.9 (2.1)	−1.1(1.4)	−27.0(28.6)

a Measurement of ability to accelerate and decelerate, pick up objects, change direction, distal coordination and navigation around obstacles. Scale score normative mean 15 (SD 5).

## 4 Discussion

In previous publications of velmanase alfa trials across pediatric and adult patients, the BOT-2 total point score was used to provide a composite measure of fine and gross motor proficiency and was used to measure efficacy and global skill acquisition across multiple domains. Musculoskeletal system abnormalities occurring in early childhood impact mobility and physical endurance throughout adolescence and adulthood [3]. Analysis of BOT-2 subtests compared to normative samples serve to better define impairment and mobility limitations in individuals of varying ages with alpha-mannosidosis and allows assessment of baseline status as well as treatment outcomes over time. Scale and age equivalent scores were used in the present analysis to enable interpretation of functional responses between patient groups with different mean ages compared to normative data. Since the analysis time was up to 48 months, assessment of changes in BOT-2 scores were based on the developmental expectations at different ages. An increase in age equivalent values for the BOT-2 indicates skill acquisition. A stable or increasing scale score also indicates skill acquisition since children in the normative comparison sample are continuing to gain skills and the reference skill set for comparison is different at every age. An increase in scale score indicates a reduction in the delay or difference between the treated group and normal healthy peers. A stable scale score indicates continued skill acquisition at a rate similar to children in the same age group in the normative sample without progression in the level of developmental delay relative to normal healthy peers.

Patients showed global developmental delays at baseline, consistent with previous assessments [5]. Subtest analysis of individual BOT-2 items captured some improvement trends with velmanase alfa, primarily in pediatric patients. At the last observation, improvements were seen in both the overall age equivalent and < 18 year scale scores for the FMP, MD, ULC and BC, indicating continued skill acquisition. FMI showed an increase for the age equivalent score (indicating continued developmental skill acquisition) but a decrease for the <18 year scale score (thus a lower rate or number of skills acquired compared with typically developing children of the same age over the same period). RSA and Balance showed no change in the overall age equivalent score, and a decrease in the <18 year scale score. Challenges with task completion due to cognitive impairment and attentional issues may have been a confounding variable.

The subtests of FMP, FMI, MD and ULC all provide a measurement of VMC. Visual motor control has a strong relationship to ability to achieve and perform reading, math and written language skills. While many patients with alpha-mannosidosis are severely limited in their abilities to perform these skills and tasks, there is a wide range of disease severity, and study suggests that children and adolescents retain potential for cognitive development [5]. Thus, any improvements can have profound impact on patient quality of life. Precision and speed of motor movements are also important for skilled performance in vocational and play activities. Limitation in mobility is a significant factor in patient reported quality of life assessments [4].

Impaired coordination is a progressive and chronic problem for patients with alpha-mannosidosis [[2], [3], [4],^19^,^20^]. Improvements in bilateral coordination as measured by the finger to nose item and the synchronized tapping items indicate possible responses to velmanase alfa. A stable score is also important as an indication of no further decline or disease progression. Although the children are not functioning at their chronological age, there was an overall reduction in challenges in dexterity and coordination and fine motor delay relative to healthy peers.

There are several overall strengths of the BOT-2 assessments. Item performance on the BOT-2 is expressed in various ways that translate into real-world functional status of patients with alpha-mannosidosis. These include measuring the amount of time to complete a task, the number of tasks completed, or the number of errors while completing the task. Although the items don't always duplicate specific daily activities, the skills required to complete the tasks correspond to typical mobility, activities of daily living, academic, and vocational demands for patients with mobility and cognitive issues. However, there are also limitations to the tests, including confounding factors that may have been present for adults resulting in limited ability to assess the BOT-2 change responses in adults compared to children. Adults had greater baseline pain as measured by the CHAQ VAS pain scores.

Other confounding factors include the limitations that cognitive impairment and attentional issues may have had on the ability of patients to complete Balance subtests. The RSA subtest also had decreased responsiveness to change due to a floor effect on the shuttle run for lower functioning patients. Finally, RSA hopping on one foot items were too difficult for many patients, thus limiting the number of items to measure an efficacy change response.

Conclusions: Patients with alpha-mannosidosis present with global developmental delay and require assistance with many activities of daily living. There was limited ability to assess the BOT-2 change responses in adults. Pediatric patients showed stability or improvement in scaled scores relative to healthy peers, indicating continued skill acquisition, which may increase independence and contribute to improved patient quality of life.

## Disclosures

DP, AL and MGC have received fees for consulting to Chiesi Farmaceutici S.p.A.; LB has received travel fees from Chiesi Farmaceutici S.p.A. and funding for clinical trials involving alpha mannosidosis; YA has no disclosures to report; JH, AL, NG received support for conducting clinical trials involving velmanase alfa; DA, SG and FC are employees of Chiesi Farmaceutici S.p.A.
